# The Cytoskeleton and Its Role in Determining Cellulose Microfibril Angle in Secondary Cell Walls of Woody Tree Species

**DOI:** 10.3390/plants9010090

**Published:** 2020-01-10

**Authors:** Larissa Machado Tobias, Antanas V. Spokevicius, Heather E. McFarlane, Gerd Bossinger

**Affiliations:** 1 School of Ecosystem and Forest Sciences, The University of Melbourne, Creswick, Victoria 3363, Australia; avjs@unimelb.edu.au (A.V.S.); gerd@unimelb.edu.au (G.B.); 2 Department of Cell and Systems Biology, University of Toronto, Toronto, ON M5S 3B2, Canada

**Keywords:** microtubules, MFA, cellulose deposition, cell wall patterning, reaction wood

## Abstract

Recent advances in our understanding of the molecular control of secondary cell wall (SCW) formation have shed light on molecular mechanisms that underpin domestication traits related to wood formation. One such trait is the cellulose microfibril angle (MFA), an important wood quality determinant that varies along tree developmental phases and in response to gravitational stimulus. The cytoskeleton, mainly composed of microtubules and actin filaments, collectively contribute to plant growth and development by participating in several cellular processes, including cellulose deposition. Studies in Arabidopsis have significantly aided our understanding of the roles of microtubules in xylem cell development during which correct SCW deposition and patterning are essential to provide structural support and allow for water transport. In contrast, studies relating to SCW formation in xylary elements performed in woody trees remain elusive. In combination, the data reviewed here suggest that the cytoskeleton plays important roles in determining the exact sites of cellulose deposition, overall SCW patterning and more specifically, the alignment and orientation of cellulose microfibrils. By relating the reviewed evidence to the process of wood formation, we present a model of microtubule participation in determining MFA in woody trees forming reaction wood (RW).

## 1. Introduction

The differentiation of a vascular cambium and secondary tissues are amongst the most important events in the evolution of higher plants and a prerequisite for the existence of woody trees. Since its first appearance in the middle Devonian (~390 Ma), secondary xylem – wood – has adapted to transport water and minerals from the roots to the shoots and to support the plant’s upright position [1]. Forest trees are important as a source of timber, pulp for paper production, biofuel and other wood products, and significant effort has been directed at functionally characterising specific genes that control wood and secondary cell wall (SCW) formation and underpin related domestication traits.

Ultimately, wood features are defined by polysaccharide-rich cell walls that determine shape, growth and rigidity of cells and tissues. Plant cell walls largely consist of cellulose, which is synthesised by cellulose synthase (CESA) proteins, integral membrane glycotransferases arranged into a unique hexagonal “rosette” structure, the cellulose synthase complex (CSC) [2]. CSCs move through the plasma membrane synthesising many individual β-1,4-glucan chains per complex that associate to form cellulose microfibrils, which are laid down into the extracellular space forming the primary cell wall (PCW) framework. Cellulose microfibrils are cross-linked to each other within a matrix formed by pectins and hemicellulose (mainly xyloglucan in PCW) [3,4]. In some cell types, including tracheary elements and xylem fibres, a lignified secondary cell wall (SCW) is deposited within the primary wall after the completion of cell expansion. The SCW has distinct types of hemicellulose (xylan and glucomannan) and a minor amount of structural proteins and enzymes when compared to the PCW [5,6]. In addition, in the SCW, the differential angle of cellulose microfibrils with respect to the long axis of a cell—a feature known as microfibril angle (MFA)—is well documented. This is especially true in the S_2_ layer, the large middle layer in a usually three-layered SCW, in which MFA is the main determinant of cell architecture and mechanical properties of fibres and tracheids [7]. While cellulose microfibrils represent the basic structural component of plant cell walls, the presence of hydrophobic lignin in SCWs results in dehydration that provides strength and resistance to negative pressure from water transport [3,8]. The organisation and/or patterning of these microfibrils and their interaction with the matrix to a large degree determine the mechanical and physiological properties of the cell wall.

The relationship between the microtubule cytoskeleton and cellulose deposition has been extensively investigated; however, much of our knowledge still relies on model organisms like Arabidopsis, while the molecular machinery behind secondary cell wall formation in woody species is largely understudied. Therefore, we reviewed aspects of the involvement of microtubules and actin filaments in secondary cell wall formation in xylary cells of woody trees and put forward a model of cellulose microfibril angle (MFA) determination in trees forming reaction wood (RW) as a response to gravitational stimulus.

## 2. MFA as a Key Feature of SCW Formation

During primary growth, cell expansion largely depends on microfibril reorientation in the PCW. Once SCW deposition commences, the cell has reached its final size and shape, and the pattern of microfibril orientation is fixed [4,9]. Green [10] hypothesised that the shape of plant cells is determined by the orientation of cortical microtubules and many studies reported the effects of microtubule disruption on cell growth [11,12,13,14,15]. For example, Pierce et al. [16] demonstrated the effect of microtubule disruption on the diameter of fibre tips in cotton (*Gossypium spp.*). Data from hypocotyl epidermal cells of Arabidopsis demonstrated that microtubules rearrange in a growth-sensitive manner as they only switch to a transversal alignment during the acceleration in growth rate [17]. However, this transverse orientation is not required to be maintained throughout the entire fast-growing phase, and once the growth rate slows, microtubules rearrange to an oblique orientation [18,19]. Preston [20] originally suggested a mathematical equation expressing the correlation between the inclination of cellulose microfibrils and the length of *Pinus* tracheids. An inverse relationship between MFA and cell length is generally accepted and has been reported in a number of studies [21,22,23]. However, reports are not consistent across the scientific literature as some authors argue that tracheid length is not related to MFA [24,25] while Evans et al. [26] demonstrated a clear correlation between MFA, density and fibre cell wall thickness. On balance, these data suggest that cell length is possibly mediated by microtubules; however, since MFA is an important feature of SCW formation, it is unlikely that it influences cell size after cell elongation has ceased.

Wood stiffness, often referred to as longitudinal modulus of elasticity (MOE), is a combined effect of wood density and MFA; MFA accounts for up to 85% of MOE variation, making it the major determinant of this important wood feature [27,28,29,30]. Tracheids or fibres in the centre of a tree, produced during the early stages of development and frequently referred to as juvenile wood, feature higher MFA and are markedly different from mature wood in strength, stability and stiffness [21,31,32,33]. Moore et al. [34] showed that 68% of the variation in MFA in *Pinus radiata* is due to radial variation, consistent with the notion that differential MOE is required during the development of a woody tree. Elasticity provided by large MFA values allows young trees to bend with the wind and avoid damage, whereas cells produced later, usually have low MFA and provide the stiffness required to support the increasing weight of the canopy [23,29]. In some investigations, MFA in the ten inner rings showed large variability between trees [21] suggesting that featuring a high MFA value during juvenile wood formation is not as critical as exhibiting the wood properties resulting from a low MFA in mature wood. In a commercial context, faster growth rates and short-rotation cropping techniques therefore often result in negative implications for wood quality due to a high proportion of juvenile wood [23].

MFA variation is also an important feature of RW, which forms in response to gravitational stimulus, caused by wind or load, where stems or branches deviate from a vertical orientation. Under such conditions, trees respond by reorienting branches, reinforcing stress points and maintaining branch angles [35,36]. In tension wood (TW), at the upper side of angiosperm branches, the tension generated results in low MFA and, hence, the longitudinal alignment of cellulose microfibrils helps to support the leaning branch. Whereas in compression wood (CW), found at the lower side of gymnosperm branches, large MFA is observed in response to compressive forces and it has been suggested to act by “pushing” the leaning branch upright [37]. Indeed, molecular dynamics simulations showed an inverse relation between MFA and MOE when compressive strength was applied [38]. Similarly, Wang et al. [39] found a negative correlation between longitudinal tensile wood properties and MFA. The wood formed at the opposite side in each case is referred to as opposite wood (OW) and it is subjected to tensile and compressive forces in gymnosperms and angiosperms, respectively. In addition, wood formed in stems growing upright is subjected solely to vertical gravitational forces with respect to the long axis of xylogenic cells and it is often referred to as normal wood (NW), featuring intermediate MFA values when compared to RW and OW [7,40,41].

## 3. Cellulose Properties and the CSC

A recent comprehensive investigation of CSC structure revealed that three CESA proteins would fit best in each triangular lobe of the rosette, totaling 18 CESAs per complex [42]. Earlier, coexpression data in Arabidopsis identified specific CESA isoforms responsible for cellulose synthesis in PCW and SCW [43,44]. The existence of six CESA classes is broadly accepted and each class corresponds to one of the six isoforms [45]. While three different classes of CESA coexisting in a CSC at a time might lead to the assumption that each isoform occupies a specific position within the complex, Nixon et al. [42] acknowledged the possibility of homomeric lobes in the rosette structure, especially since all PCW-CESAs are able to homodimerise [46]. In this context, Zhang et al. [47] studied the stoichiometry of CESAs in aspen (*Populus tremula*) SCW CSCs and found a 3:2:1 ratio for CESA8:CESA4:CESA7 in normal wood that changed to 8:3:1 in TW, also coinciding with an increase in crystalline cellulose microfibril diameter. These results support alternative CSC models and suggest that homomeric CESA8 complexes might be the main CESA responsible for cellulose biosynthesis during TW formation. This implies that the composition of the CSC in developing xylem fibres potentially plays specific roles with consequences for cellulose microfibril properties.

While MFA in SCWs is particularly relevant during wood formation in tree species, much of our current knowledge still relies on experimental model organisms and systems. Arabidopsis mutants with aberrant SCW patterning, for example, feature changes in MFA [48] and differential composition of the CSC results in alterations in cellulose microfibril polymerisation [49,50]. Even though CSC plays the same role in synthesising cellulose both in primary and secondary walls, variations in the composition and structure of CSCs appears to impart structural variations in cellulose microfibrils [49]. Nevertheless, our current understanding remains sketchy and more studies investigating the complex molecular relations with the CSC during SCW deposition are required.

## 4. Cytoskeleton Roles in SCW Biosynthesis

The cytoskeleton is an intracellular framework present in all cellular organisms composed of filamentous polymers characterised by high dynamicity. In plant cells, the main components of the cytoskeleton are microtubules and actin filaments that form an intricate array in plant cells and collectively assume key roles in cell division, cytoplasmic streaming and organelle transport. Both elements, particularly microtubules, are touted to participate in cell wall biosynthesis with different degrees of involvement. A number of studies have investigated the cytoskeleton dynamics in xylary cells undergoing SCW deposition employing different image techniques [51,52,53]. Microfilaments align longitudinally in fusiform cambial cells and their derivatives in both angiosperms and gymnosperms, while microtubules switch from a transverse to an oblique orientation during SCW formation [54,55]. Furthermore, the cytoskeleton arrangement differs in fibres depositing a G-layer where both microfilaments and microtubules are arranged axially [54]. Finally, microfilaments and microtubules associate with pit formation in tracheids and fibres [54,55]. This review, therefore, investigates and summarises our current understanding of the role played by the cytoskeleton in guiding SCW deposition in xylary cells before proposing a mechanistic molecular model for MFA determination in response to gravitational stimulus.

### 4.1. Microtubules Guide the CSC and Play a Role in SCW Patterning

The primary components of microtubules are globular proteins called tubulins that form heterodimers comprised of α and a β monomers that bind head to tail, forming protofilaments [56]. Commonly, 13 protofilaments bind together to form the hollow cylindric structure of the microtubule that undergoes stochastic changes between growing and shrinking phases based on GTP hydrolysis, resulting in the well-recognised dynamic microtubule network [57]. In contrast to other eukaryotes, plant cells do not have a well-defined microtubule organising centre, which contributes to their high responsiveness to environmental triggers [58]. In plants, microtubules are arranged into super-structures such as the preprophase band, the spindle and the phragmoplast during cell division [58,59] and the interphasic cortical array, where microtubules organised into a parallel array associated with the plasma membrane, which is believed to regulate the cellulose deposition pattern [10].

The relationship between microtubules and cellulose deposition has been studied by genetic and pharmacological techniques, and, more recently, live cell imaging has been used to assess the dynamics of these structures. Inhibition of cortical microtubule organisation or cellulose microfibril deposition by pharmacological and genetic approaches suggests that cortical microtubules control the movement of CSCs. Baskin et al. [60] reported variable cellulose microfibril orientation with increasing concentrations of oryzalin, a microtubule polymerisation inhibitor, at the cellular level but not at a local subcellular level (<10 µm^2^). Paredez et al. [61] described two oryzalin hypersensitive Arabidopsis mutants of PROCUSTE1 and KORRIGAN, genes previously shown to be involved in cellulose biosynthesis, indicating a bidirectional flow of information between microtubules and CESAs. Paredez et al. [62] characterised CSC behaviour in PCWs, demonstrating that microtubules share spatiotemporal locations with CSCs that move along tracks delineated by them. Later, Watanabe et al. [63] demonstrated the existence of the same mechanism in SCW. These findings provide fundamental evidence for interactions between microtubules and the CSC, consistent with the hypothesis that the cortical microtubule organisation in plant cells, directly or indirectly, offers cues for cellulose synthesis.

Two models have been proposed to explain the alignment between cellulose microfibrils and microtubules: the direct guidance model and the constraint or bumper model [64,65]. These models differ with respect to the requirement of a physical linker between CSCs and cortical microtubules. Following the direct guidance model, many microtubule-associated proteins (MAPs) were investigated for their potential roles as linkers between CSC and microtubules. The fragile fibre 1 (FRA1) kinesin motor protein, for example, was suspected to play a role in CSC and microtubule binding based on the aberrant cellulose microfibril deposition observed in *fra1* mutants [66]. Further characterisation demonstrated the function of this protein as a bona fide motor protein that binds to microtubules; however, it remains questionable if FRA1 is indeed required to guide CSC movement since in vitro it moves in a unidirectional fashion much faster than CESA [62,67]. Also, the activity of Cellulose Synthase Interactive Protein 1 (CSI1) and *pom-pom2* mutants, which are allelic, were identified to physically link microtubules and CSC [68,69]. CSI1/POM2 interact with SCW CESAs in a similar way as with PCW CESAs and its downregulation causes abnormal SCW deposition [48]. Cells within stems of *pom2-4* mutants showed significantly higher MFA when compared to wild-type [48], demonstrating that correct interaction between CSC and microtubules is necessary to determine cellulose microfibril orientation. Kesten et al. [70] showed that the companion of cellulose synthase 1 (CC1) is part of the CSC and links to microtubules, playing a critical role in “re-stablishing” the microtubule array after perturbations caused by salt stress. Conversely, in accordance with the bumper model, a recent study using near-TIRF microscopy and high-throughput particle-tracking analysis concluded that microtubules affect CSC speed by mechanisms that are independent of direct physical association [71]. These observations and the fact that CSCs rapidly recover after microtubule organisation disruption by oryzalin [48,62] suggest that both models are correct and that it is likely that physical association of CSCs and microtubules is critical at the start of cellulose synthesis, but guidance is not strictly necessary to ensure continuous movement in straight lines during the late phases of cell development.

Microtubule organisation is also believed to play a role in determining SCW pattern in xylem cells. The SCW pits present in metaxylem cells appear to be guided by localised microtubule depolymerisation. ROP11, a plant-specific small GTPase from the Rac/Rho family, localises at the plasma membrane of metaxylem differentiating cells within pit areas and recruits Microtubule Depletion Domain 1 Protein (MIDD1), which is responsible for microtubule depolymerisation from these cellulose-depleted regions [72]. Simultaneously, cell wall growth is promoted at pit boundaries through the ROP-BDR-WAL-actin pathway (see below) allowing for precise control of bordered pit formation [73]. In addition, Cortical Microtubule Disordering Protein 1 (CORD1) affects microtubule organisation, branching angle and microtubule attachment to the plasma membrane, resulting in abnormally enlarged SCW pits [74]. Other MAPs belonging to MAP20 and MAP70 families were also suggested to play a role in cellulose synthesis and SCW patterning in xylem cells [75,76]. Microtubules undoubtedly play a role in cell wall biosynthesis, and reasonably exert influence on MFA; however, further research is needed to elucidate the exact nature of this control at the molecular level.

### 4.2. Actin Filaments and Microtubules Act Together to Deliver CSC to the Plasma Membrane

The actin cytoskeleton is composed of stranded filaments of globular actins and its main functions in plant cells are related to cellular growth, cytoplasmic streaming, cell division and organelle movement [77]. Monomers of globular actin (G-actin) self-assemble into filaments in a similar way as tubulin dimers do. However, in contrast to the GTP dependent assembly of microtubules, this process is ATP dependent. In terms of organisation, the actin cytoskeleton can exhibit a much more diverse organisation when compared to microtubules. During interphase, the actin cytoskeleton appears to assume two different arrangements: a cortical array and subcortical axial bundles that are established and maintained by actin-binding proteins (ABPs) and actin-related proteins (ARPs) [77,78]. The most reported function of actin filaments in all cellular organisms is to control cell polarisation, as has been especially well established for tip-growing plant cells, such as pollen tubes and root hairs [78]. Notably, relative to microtubules, actin roles in cell wall biosynthesis have been discovered at a slower pace.

**Figure 1:**
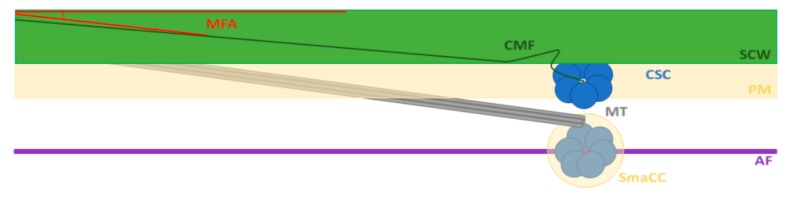
Model of cytoskeleton roles in MFA determination during xylem cell development. SmaCCs movement is affected by actin filaments and CSCs are delivered to the plasma membrane at SCW depositing sites marked by bundles of microtubules. Microtubules also influence the angle at which cellulose microfibrils are deposited within the cell wall. AF, actin filament; CMF, cellulose microfibril; CSC, cellulose synthase complex; MFA, microfibril angle; MT, microtubule; PM, plasma membrane; SCW, secondary cell wall; SmaCC, small CESA-containing compartment.

Kobayashi et al. [79] were amongst the first to notice an influence of the actin cytoskeleton on cellulose deposition in SCWs; cultured Zinnia mesophyll cells differentiated into tracheary elements and treatment with the actin inhibitor cytochalasin B, produced abnormal cell wall patterning. Later, Sampathkumar et al. [78] using both pharmacological and genetic approaches, reported that defects in the organisation of the actin cytoskeleton resulted in cellulose deficient Arabidopsis seedlings with cell walls of variable thickness. CSC distribution in the plasma membrane likely depends on actin-based long-distance transport since CSC delivery rate is limited, and proximity is increased when actin cytoskeleton organisation is disrupted [78]. Therefore, fast and even distribution of CSCs to the plasma membrane depends on actin filaments [80]. CSC-containing organelles are longitudinally transported by actin cables around the cell to sites marked by transverse actin filaments and CSCs are then incorporated into the plasma membrane and kept at SCW depositing sites by bundles of microtubules [81]. These organelles have been characterised as small CESA-containing compartments (SmaCCs) and Golgi and CSC insertion are confined to SCW thickenings associated with microtubule bands [63]. To further test if actin filaments, and not microtubules, mark the CESA delivery sites, Gutierrez et al. [82] treated seedlings with latrunculin B to disassemble the actin array. They found severe disruption in CESA distribution with many cells showing areas completely depleted, even though this treatment did not directly disrupt CESA delivery to the plasma membrane. Taken together, the results of these two studies suggest that distribution of Golgi, Golgi-independent and Golgi-associated SmaCCs by actin cables and filaments are required for the correct global positioning of the CSC whereas microtubules act on a smaller scale by positioning CSCs once they are in the plasma membrane (Figure 1).

F-actin also plays an important role in patterned cell wall deposition in Arabidopsis metaxylem. Sugiyama et al. [73] demonstrated that during pit formation in the SCW of xylem vessels, ROP11 and/or other ROPs recruit wallin (WAL) to the plasma membrane via the boundary of ROP domain 1 (BDR1) and BDR3. WAL localises at pit boundaries promoting actin assembly and cell wall ingrowth [73]. This regulatory pathway has opposite effects on SCW growth in comparison to the ROP-MIDD1-microtubule pathway [72]. Together they play a crucial role in efficient water transport by allowing tight control of pit formation during SCW deposition in xylem vessels.

The seemingly coordinated actions of microtubules and actin filaments during cell wall synthesis and other cellular processes imply a level of direct or indirect communication between these two cytoskeleton components. Live cell imaging of actin filaments and microtubules clearly showed a coincidence between the arrays and interdependence for reassembly after drug treatment [83], indicating interactions between microtubules and actin filaments either directly or via associated proteins. Indeed, some proteins classically associated with microtubule regulation interact with actin filaments. This is the case for Arabidopsis microtubule associated protein 18 (MAP18) and microtubule destabilising protein 25 (MDP25) and several conventional microtubule motors like kinesin-like proteins [84,85,86,87]. Conversely, conventional actin-binding proteins (ABPs) have also been found to interact with microtubules [88,89,90]. Besides the intercommunication between the two arrays, the cytoskeleton also interacts with the cell wall via transmembrane proteins that possess an extracellular cell wall- and an intracellular cytoskeleton-binding domain in what is called the cytoskeleton-plasma membrane-cell wall continuum [91]. This continuum is presumably responsible for transmitting cell wall perturbations to the cytoskeleton resulting in reorganisation. In this respect, Tolmie et al. [92] reported an increase in actin network stability in plasmolysed cells or cells treated with isoxaben, a cellulose synthesis inhibitor, and normal dynamics were recovered in re-hydrated cells, demonstrating changes in actin cytoskeleton dynamics were highly correlated with cell wall disruption. In combination, these findings suggest a role of the plant cytoskeleton in regulating cell wall biosynthesis by determining cellulose deposition sites and cellulose microfibril orientation through the cytoskeleton-plasma membrane-cell wall continuum.

## 5. Molecular Control of MFA

The orientation of cellulose microfibrils within the cell wall determines to a large degree cell architecture and mechanical properties with significant implications for plant development [22,29]. The molecular machinery behind MFA determination is still unclear; however, many quantitative trait loci (QTL) have been identified for wood and fibre properties, including MFA [93,94,95], and some genes have been identified as candidates [95,96,97,98,99]. Reaction wood (RW) has proved to be a useful model system in attempts to better understand the molecular basis of wood formation [100] and several studies have published transcriptomes of angiosperm and gymnosperm RW [101,102,103]. A large number of genes were reported to be highly expressed in RW, among them some encoding arabinogalactan proteins (AGPs), fasciclin-like arabinogalactan proteins (FLAs) and α and β-tubulins [102,104,105,106,107,108].

TW, developed on the upper side of angiosperms branches, is characterised by extremely low MFA values and this is often associated with upregulation of many of cytoskeleton component and secondary wall formation genes, including those listed previously [41,101,104,105,106,108,109,110,111,112]. Accordingly, Li et al. [113] found the same genes overexpressed in high stiffness wood in *Pinus radiata*, which also has lower MFA when compared to low stiffness wood. Furthermore, Li et al. [114] compared gene expression in mature wood of the same species with juvenile wood featuring a 10 larger MFA and found both α- and β-tubulin genes were upregulated in mature wood, once more associating high expression levels of these genes with low MFA. In contrast, some studies linked high expression of tubulin and arabinogalactan genes in CW of loblolly pine, maritime pine and *Chamaecyparis obtusa* Siebold and Zucc, to large MFA [102,115,116,117,118]. Changes in MFA, be it due to RW formation, stress response or wood maturation, involve coordinated changes in gene expression to produce context-specific outcomes. Research results published over the last decade shed light on the roles of many genes in controlling this important feature; however, functional confirmation of their collective action to modulate MFA remains to be documented in most cases. Here we discuss the involvement of several genes in MFA determination in different species and summarise their combined action in a model of the molecular control of cellulose orientation in woody trees forming RW.

### 5.1. Arabinogalactans

The Arabinogalactanprotein (AGP) family is characterised by highly glycosylated hydroxyproline-rich glycoproteins (HRGPs) mainly expressed in plant cell walls. In general, AGPs are expressed in various organs and tissues, but individual members with different core proteins exhibit organ-, tissue- and/or developmental-specificities [119]. Because of their location in the cell wall and the presence of a predicted glycophosphotidyl inositol (GPI) anchor domain in many AGPs, which would localise them to the outer leaflet of the plasma membrane, it was suggested that their function is to transmit information between the cell wall and cytoplasm [120]. AGPs are believed to play an important role in tracheid differentiation [121] and pharmacological and genetic studies provide strong evidence for a link between AGP and microtubules [122,123,124]. The existence of lipid rafts in the plasma membrane or a third protein or protein complex has been suggested as physical linkers between AGPs and the microtubule cortical array [123,125]. Alternatively, Driouich and Baskin [120] proposed a model in which diffuse AGPs (not possessing GPI) interact with receptor kinases and another machinery of signal transduction on the plasma membrane that leads to microtubule disorganisation. Indeed, other GPI-anchored proteins have been implicated in receptor-like kinase trafficking from within the cell to the plasma membrane, possibly as cofactors or chaperones for these receptors [126]. Considering their position in the plant cell and how microtubules and arabinogalactan proteins seem to communicate, it can be speculated that cortical microtubules and the CSC might associate via AGPs; however, further work is needed to demonstrate the actual effects of AGP on cellulose deposition and microfibril orientation. 

The fasciclin (FAS) domain is a cell adhesion domain found in proteins of both eukaryotes and prokaryotes and, despite its considerable level of conservation, it assumes a number of different functions across kingdoms [127,128]. In plants, FAS-containing proteins belong to the superfamily of AGPs and form FAS-like arabinogalactan proteins (FLAs) [129]. Twenty-one FLAs have been described in Arabidopsis [130], 27 in rice [131], 19 in cotton [132], 18 in *Eucalyptus grandis* [133] and 15 in poplar [104]. *FLA* genes are classified in four groups according to the number of FAS domains [130] and, specifically, a single-FAS group (group A) shows stem-preferential expression, particularly in developing xylem cells [107]. This expression of group-A *FLA*s was reported to be 3- to 27-fold higher on the upper side of eucalypt branches compared to lower branch wood [106,133] suggesting that *FLA*s might have a function in SCW biosynthesis in RW and in determining stem biomechanical properties.

Genetic studies revealed FLA effects on cellulose biosynthesis and deposition. Persson et al. [134] reported high levels of co-regulation of *FLA11* and *12* with secondary cell wall-associated *CESA4, 7* and *8* in Arabidopsis and a 2 increase in MFA was found in At*fla11*/*fla12* Arabidopsis mutants [107]. Dahiya et al. [135] reported a putative homolog of *AtFLA11* in *Zinnia* (*ZnFLA11*) to be exclusively expressed in metaxylem with reticulate SCW thickening. Furthermore, the Arabidopsis *FLA4*, also called *SALT OVERLY SENSITIVE 5* (*SOS5*), was shown to act on a pathway that regulates the synthesis of cellulose in Arabidopsis roots similarly to FEI1/FEI2 receptor-like kinases [136]. Similarly, Huang et al. [137] identified the AtVRLK1 (*Vascular-Related Receptor-Like Kinase1*), that is specifically expressed in xylem cells undergoing SCW differentiation and seems to promote cell elongation and restrain cell wall thickening in those cells. In trees, a correlation between *FLA* expression, MFA and wood properties was established [104,106,138,139] and MacMillan et al. [133] demonstrated that overexpression of *EfrFla* genes led to a 3 reduction in MFA in eucalypt fibres and biomechanically impacted tobacco stem cells. Adhesion domains (FAS) may function by binding structural components of the cell wall and the extracellular matrix or by interacting with extracellular signals to alter microtubule orientation [133]. In poplar, *FLA*s were specifically linked to the formation of the gelatinous-layer of TW cells, probably as a result of tension generation. [139]. Together, these results suggest a role of FLA proteins in SCW formation.

### 5.2. Tubulins

High expression levels of tubulin genes, encoding the primary components of microtubules, are also associated with RW formation. Oakley et al. [140] assessed transcript expression levels in cambial tissue of bent stems in poplar and found tubulin genes, *TUA1*, *TUA5*, *TUB9* and *TUB15*, to be specifically up-regulated 2- to 4-fold when compared to cambial tissue of upright stems. In plants, tubulins are encoded by multigene families and the expression of different isoforms is tissue specific and varies throughout plant development, with tissue-preferential clusters grouping separately in phylogenetic analyses [140,141]. For instance, the Arabidopsis genome contains six *TUA*s, one of them specifically expressed in pollen tubes, and nine *TUBs* with differential expression in roots, leaves and floral tissue [142,143]. Rice (*Oryza sativa*) also has a pollen-specific β-tubulin isoform and another seven which are differentially expressed in vegetative tissue [144]. In cotton (*Gossypium hirsutum*), five *TUA*s are expressed in elongating fibres and only two remain highly expressed once SCW deposition is initiated [145]. In Poplar, the TUA family has eight members and the TUB family has undergone significant expansion and contains 20 members as ten pairs of highly homologous *TUB*s [140]. The large number of tubulin isoforms poses the question why all of them are maintained. The multi-tubulin hypothesis interprets tubulin diversity as a requirement for differential microtubule formation [146]. Conversely, tubulin gene redundancy might ensure the expression of a fundamental protein [141]. Besides the large number of isoforms, it is believed that post-translational modifications (PTM) in tubulins mark microtubules with distinct stability and association with MAPs and motor proteins, thereby altering their sensitivity to microtubule-disrupting drugs [141]. Hence, selective expression of tubulin genes and PTM accumulation could, collectively, finely adjust microtubule assembly and/or dynamics in specific tissues to perform required functions [147].

Few studies have uncovered functional links between tubulins and MFA. Spokevicius et al. [40] established that β-tubulin affects cellulose microfibril orientation in fibre SCWs of young eucalypt trees. In their study, phenotype-based evidence indicates that downregulation of an *Eucalyptus grandis* β-tubulin gene (*EgrTUB1*) causes a significant increase in MFA in transgenic fibres, suggesting that this β-tubulin isoform is directly involved in determining cellulose microfibril orientation during xylogenesis, possibly via changes to microtubule structure. Swamy et al. [148] studied post-translational modifications in poplar TUA1 but did not find any effects on MFA of transgenic trees. This study demonstrated that non-cellulosic polysaccharides—deposited early during cell wall biosynthesis—are more sensitive to this type of tubulin manipulation in wood cells than cellulose content and its organisational features like MFA or crystallinity. Therefore, these efforts made it evident that modifications of microtubule organisation by targeting tubulins have the potential to significantly affect the mechanical properties of plant cell walls. Nevertheless, studies attempting to understand how microtubules coordinate microfibril orientation directed towards specific tubulin roles are still elusive and virtually nothing is known about the molecular mode of action of these specific protein isoforms in MFA determination.

### 5.3. Other Cell Wall-Related Genes

Other genes known to have effects on cell wall formation and influence MFA include the *KORRIGAN* (*KOR*) gene. *KOR* was isolated from the Arabidopsis mutant *kor1-1*, which is characterised by abnormal PCW formation in the absence of light [149], and further characterisation demonstrated that *KOR* plays roles in cytokinesis, cell elongation and cellulose synthesis [150] and influences pectin metabolism [151]. Despite the majority of roles having been described in PCW synthesis, Szyjanowicz et al. [152] report *KOR* functions also in the SCW. In poplar, two families of glycosyl hydrolase genes were shown to be similar to Arabidopsis *KOR* [153] and these genes were found to be up-regulated in cells undergoing secondary wall formation [112,154]. Maloney and Mansfield [150] demonstrated architectural alterations in poplar wood due to downregulation of *PaxgKOR* with transgenic trees exhibiting lower cellulose content, changes in cellulose composition, an increase in cellulose crystallinity and significantly lower MFA. 

Finally, xylan-acting enzymes were found to be upregulated during xylem SCW formation in aspen and to affect MFA and other aspects of plant development. Xylans, the main hemicellulose of SCW, are polymers with a β-1,4-D-xylopyranose backbone [155] and the downregulation of an aspen gene encoding the endo-1,4-β-xylanase belonging to the glycoside hydrolase family 10 (*PtxtXYN10A*) resulted in a clear reduction of MFA of transgenic fibres, indicating a role for this gene in orientating cellulose microfibrils in secondary walls [156].

### 5.4. A Molecular Model for MFA Alterations in Response to Gravitational Stimulus

While the roles of *KOR* and *XYN* have not been demonstrated in other species, *TUB*s and *AGP*s, *FLA*s among them, have shown a consistent expression profile in several studies across species and deserve to be more carefully examined here to understand their combined actions during cellulose biosynthesis. Stress has been shown to either up- or downregulate AGP expression [157], with some AGPs being upregulated in TW while others are downregulated [106]. The perception of gravity, a mechanical stimulus in nature, and a plant’s capability to respond to it are not fully understood. However, the roles of different cell wall components and the secondary messenger calcium (Ca^2+^) have been proposed [158,159]. Due to their position and structure, AGPs are strong candidates to function as mechanical sensors, sensing both tension and compression within the cell wall [160]. Moreover, AGP can bind and release Ca^2+^ in the cell under certain conditions [161]. Ca^2+^ plays critical roles during stress response activating important signal-mediators such as phosphatases and phospholipases [162]. The rapid activation of phospholipase D (PLD) during stress cleaves the GPI anchor and releases AGP from the plasma membrane into the cell wall [157] and might function as another signaling molecule in the cascade of events that culminates in the transcription of *AGP* genes. In addition, one of the products of PLD, phosphatidic acid (PA), is known to activate the mitogen-activated kinase protein (MPK6) which can phosphorylate the microtubule associated protein MAP65-1 leading to destabilisation of the microtubule array in dividing cells [163]. Conversely, PA binds to MAP65-1 promoting microtubule reorganisation [164]. MAP65-1 ensures array stabilisation by promoting microtubule bundling [165] and it plays an important role in different types of stress, such as salt and cold response [70,166,167,168]. Ca^2+^ also binds to the protein phosphatase PP2A and one Arabidopsis gene belonging to its regulatory subunit B” is involved in microtubule nucleation [169], possibly by regulating the Augmin complex which, in turn, recruits the γ-tubulin-containing ring complex (γTuRC) and promotes microtubule nucleation [170].

Another important stress signaling mediator is auxin. The plasma membrane protein family PIN-FORMED (PIN) acts in auxin efflux and is important for intercellular auxin signaling. PIN1 has been reported to localise to membranes adjacent to cell walls subjected to the highest stress and correlates to cortical microtubule array orientation in Arabidopsis shoot apical meristems [171]. While changes in auxin balance seem not to contribute to RW formation [172], they activate the ROP6-RIC1 pathway through auxin binding protein 1 (ABP1) [173]. RIC1 binds to the p60 subunit of the Arabidopsis katanin 1 (KTN1), a microtubule severing protein involved in mechanical stress response [174,175,176]. Finally, microtubule nucleation and severing of nascent microtubules followed by depolymerisation of mother-microtubules are key steps in a cortical microtubule array shift from a transverse to an oblique reorientation [177].

**Figure 2:**
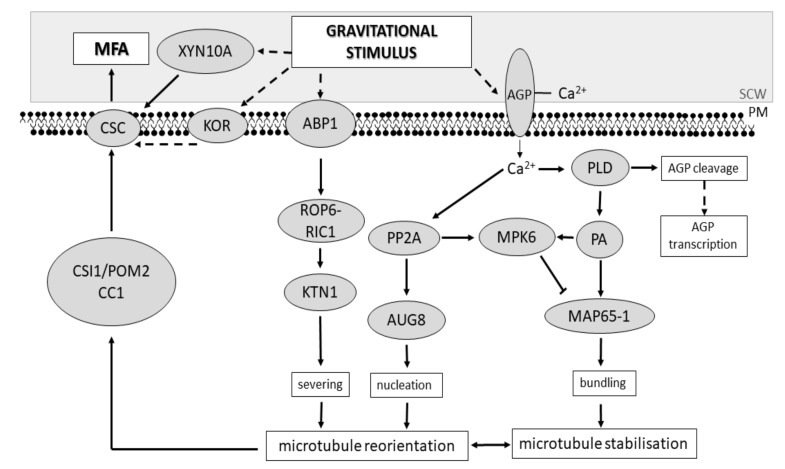
Model of MFA determination in response to gravitational stimulus. Gravitational stimulus is potentially sensed by AGP proteins, which could result in the release of calcium into the cell. Ca2+ activates PLD and PP2A that act on downstream targets and promote cortical microtubule array reorientation and stabilisation. Microtubules interact with the CSC through CSI1/POM2 and CC1 to determine MFA. ABP1, auxin binding protein 1; AGP, arabinogalactan protein; AUG8, AUGMIN subunit 8; CC1, companion of cellulose synthase 1; CSC, cellulose synthase complex; CSI1/POM2, cellulose synthase interacting 1; KOR, korrigan; KTN1, katanin 1; MAP65-1, microtubule associated protein 65-1; MPK6, mitogen activated kinase 6; PA, phosphatidic acid; PLD, phospholipase D; PM, plasma membrane; PP2A, protein phosphatase 2A; ROP6, plants Rho-related GTPase; RIC1, ROP-interactive CRIB motif-containing protein 1; SCW, secondary cell wall; XYN10A, endo-1,4-β-xylanase glycoside hydrolase 10. Solid lines indicate experimentally determined interactions, while dashed lines indicate hypothesised connections.

Due to the gene expression profile of RW, we hypothesise that the perception of gravitational stimulus by AGPs, PINs, and other proteins causes Ca^2+^ influx that activates PP2A. In turn, PP2A acts on promoting nucleation of microtubules through the action of AUG8. Branched nucleation followed by severing of newly formed microtubules promoted by KTN1 results in reorientation of the microtubule array. Moreover, other Ca^2+^ targets include PLD, which acts on microtubule associated proteins (MAPs) responsible for promoting microtubule stabilisation through bundling of microtubules. Shifts in microtubule orientation of differentiating wood cells lead to MFA changes during RW formation (Figure 2). Nevertheless, further work is required to elucidate the fine-tuning mechanisms responsible for correct tissue and function specific cellulose microfibril deposition angles.

## 6. Conclusions

The cytoskeleton plays an important role in determining CSC delivery sites during SCW deposition, and, specifically in xylary cells, it has been demonstrated to be critical for proper microfibril orientation. Both microtubules and actin filaments are involved in patterning secondary wall deposition of xylem cells, which ensures essential features of wood related to upright support and water transport. These features are of special interest particularly in woody trees because they can impact wood quality.

Studies in Arabidopsis have been essential in aiding our understanding of the roles of cytoskeleton in xylem cell development. The roles of microtubules and actin filaments coordinated by MAPs and ABPs within the cytoskeleton-plasma membrane-cell wall continuum have been revealed, but these only form part of a robust regulatory pathway that encompasses environmental perception and signal translation. Demonstrations of similar processes in woody trees are still wanting. In their absence and in utilising all available evidence, we have put forward a hypothesis for how microtubules might participate in MFA determination in response to gravitational stimulus, which can be used as a molecular model for future studies that will investigate how tubulins interact with other molecular components to shape xylary cells in response to environmental changes in woody trees.
