# Erratum: The Cytoskeleton and Its Role in Determining Cellulose Microfibril Angle in Secondary Cell Walls of Woody Tree Species. *Plants* 2020, *9*, 90.

**DOI:** 10.3390/plants9020255

**Published:** 2020-02-17

**Authors:** Plants Editorial Office

**Affiliations:** MDPI, St. Alban-Anlage 66, 4052 Basel, Switzerland; plants@mdpi.com

The Editorial Office of *Plants* wants to make the following corrections to the paper by Tobias, L.M., et al. (2020) [1]:

On Page 5, move “(see below)” to after [72].

On Page 10, delete “, possibly by regulating the Augmin complex which, in turn, recruits the -tubulin-containing ring complex (TuRC) and promotes microtubule nucleation [170]”.

On Page 10, delete “through the action of AUG8”.

On Page 10, revise Figure 2 and delete AUG8, AUGMIN subunit 8 in the figure caption.

In the References, delete Reference [170] and reorder all references.

The manuscript will be updated and the original will remain online on the article webpage.

We would like to apologize for any inconvenience caused.
